# Visualization and Measurement of ATP Levels in Living Cells Replicating Hepatitis C Virus Genome RNA

**DOI:** 10.1371/journal.ppat.1002561

**Published:** 2012-03-01

**Authors:** Tomomi Ando, Hiromi Imamura, Ryosuke Suzuki, Hideki Aizaki, Toshiki Watanabe, Takaji Wakita, Tetsuro Suzuki

**Affiliations:** 1 Department of Virology II, National Institute of Infectious Diseases, Tokyo, Japan; 2 Graduate School of Frontier Sciences, The University of Tokyo, Tokyo, Japan; 3 The Hakubi Center and Graduate School of Biostudies, Kyoto University, Kyoto, Japan; 4 Hamamatsu University School of Medicine, Department of Infectious Diseases, Hamamatsu, Japan; Fundación Instituto Leloir-CONICET, Argentina

## Abstract

Adenosine 5′-triphosphate (ATP) is the primary energy currency of all living organisms and participates in a variety of cellular processes. Although ATP requirements during viral lifecycles have been examined in a number of studies, a method by which ATP production can be monitored in real-time, and by which ATP can be quantified in individual cells and subcellular compartments, is lacking, thereby hindering studies aimed at elucidating the precise mechanisms by which viral replication energized by ATP is controlled. In this study, we investigated the fluctuation and distribution of ATP in cells during RNA replication of the hepatitis C virus (HCV), a member of the *Flaviviridae* family. We demonstrated that cells involved in viral RNA replication actively consumed ATP, thereby reducing cytoplasmic ATP levels. Subsequently, a method to measure ATP levels at putative subcellular sites of HCV RNA replication in living cells was developed by introducing a recently-established Förster resonance energy transfer (FRET)-based ATP indicator, called ATeam, into the NS5A coding region of the HCV replicon. Using this method, we were able to observe the formation of ATP-enriched dot-like structures, which co-localize with non-structural viral proteins, within the cytoplasm of HCV-replicating cells but not in non-replicating cells. The obtained FRET signals allowed us to estimate ATP concentrations within HCV replicating cells as ∼5 mM at possible replicating sites and ∼1 mM at peripheral sites that did not appear to be involved in HCV replication. In contrast, cytoplasmic ATP levels in non-replicating Huh-7 cells were estimated as ∼2 mM. To our knowledge, this is the first study to demonstrate changes in ATP concentration within cells during replication of the HCV genome and increased ATP levels at distinct sites within replicating cells. ATeam may be a powerful tool for the study of energy metabolism during replication of the viral genome.

## Author Summary

ATP is the major energy currency of living cells. Replication of the virus genome is a physiological mechanism that is known to require energy for operations such as the synthesis of DNA or RNA and their unwinding. However, it has been difficult to comprehend how the ATP level is regulated inside single living cells where the virus replicates, since average ATP values in cell extracts have only been estimated using existing methods for ATP measurement. ATeam, which was established in 2009, is a genetically-encoded Förster resonance energy transfer (FRET)-based indicator for ATP that is composed of a small bacterial protein that specifically binds ATP sandwiched between two fluorescent proteins. In this study, by applying ATeam to the subgenomic replicon system, we have developed a method to monitor ATP at putative subcellular sites of RNA replication of the hepatitis C virus (HCV), a major human pathogen associated with liver disease, in living cells. We show here, for the first time, changes in ATP concentrations at distinct sites within cells undergoing HCV RNA replication. ATeam might open the door to understanding how regulation of ATP can affect the lifecycles of pathogens.

## Introduction

Adenosine 5′-triphosphate (ATP) is the major energy currency of cells and is involved in a variety of cellular processes, including the virus life cycle, in which ATP-dependent reactions essential for virus multiplication are catalyzed by viral-encoded enzymes or complexes consisting of viral and host-cell proteins [1]. However, the lack of a real-time monitoring system for ATP has hindered studies aimed at elucidating the mechanisms by which cellular processes are controlled through ATP. A method for measuring ATP levels in individual living cells has recently been developed using a genetically-encoded FRET-based indicator for ATP, called ATeam, which employs the epsilon subunit of a bacterial F_0_F_1_-ATPase [2]. The epsilon subunit has several theoretical advantages for use as an ATP indicator; i) small size (14 kDa), ii) high specific binding to ATP, iii) ATP binding induces a global conformational change and iv) ATP hydrolysis does not occur following binding [3]–[5]. The affinity of ATeam for ATP can be adjusted by changing various amino acid residues in the ATP-binding domain within the subunit. ATeam has enabled researchers to examine the subcellular compartmentation of ATP as well as time-dependent changes in cellular ATP levels under various physiological conditions. For example, the ATeam-based method has been used to demonstrate that ATP levels within the mitochondrial matrix are lower than those in the cytoplasm and the nucleus [2].

Hepatitis C virus (HCV) infects 2–3% of the world population and is a major cause of chronic hepatitis, liver cirrhosis and hepatocellular carcinoma [6]–[8]. HCV possesses a positive-strand RNA genome and belongs to the family *Flaviviridae.* A precursor polyprotein of ∼3000 amino acids is post- or co-translationally processed by both viral and host proteases into at least ten viral products. The nonstructural (NS) proteins NS3, NS4A, NS4B, NS5A and NS5B are necessary and sufficient for autonomous HCV RNA replication. These proteins form a membrane-associated replication complex (RC), in which NS5B is the RNA-dependent RNA polymerase (RdRp) responsible for copying the RNA genome of the virus during replication [9], [10]. NS3, in addition to its protease activity, functions as a viral helicase capable of separating duplex RNA and DNA in reactions fuelled by ATP hydrolysis [11], [12]. Consistent with other positive-strand RNA viruses, replication of HCV genomic RNA is believed to occur in membrane-bound vesicles. NS3-NS5B proteins, together with several host-cell proteins, form a membrane-associated RC. The HCV RC is localized to distinct dot-like structures within the cytoplasm of HCV replicating cells and can be detected in detergent-resistant membrane structures [13].

In this study, we first used capillary electrophoresis-time-of-flight mass spectrometry (CE-TOF MS) and the original ATeam method to determine ATP levels in cells infected with HCV or replicating HCV RNA. Using these methods, together with an ATP consumption assay, we demonstrated that ATP is actively consumed in cells in which viral RNA replicates, leading to a reduction in cytoplasmic ATP compared to parental cells. To further understand the fluctuation and distribution of ATP in HCV replicating cells, we developed a system to monitor ATP at putative subcellular sites of HCV RNA replication in single living cells by applying ATeam technology to the subgenomic replicon system. Our results show that, in viral RNA-replicating cells, ATP levels are elevated at distinct dot-like structures that may play a supportive role in HCV RNA replication, while cytoplasmic levels of ATP decrease.

## Results

### The concentration of ATP is reduced in HCV-infected cells

**Figure 1:**
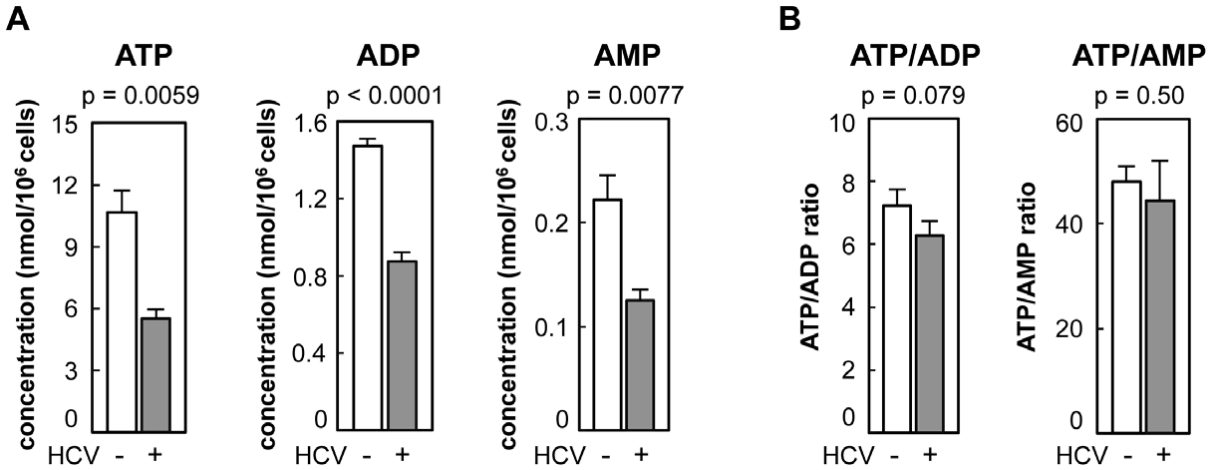
Levels of adenosine nucleotides in HCV-infected and non-infected Huh-7 cells determined by CE-TOF MS. (A) ATP levels were reduced in HCV-infected cells. ATP, ADP, and AMP metabolites in Huh-7 cells with (gray bars) and without (open bars) HCV infection were measured by CE-TOFMS. (B) Ratios of ATP/ADP and ATP/AMP were calculated from the results depicted in (A). All data are presented as means and standard deviation (SD) values for three independent samples. Statistical differences between HCV-infected and non-infected cells were evaluated using Student's *t*-test.

As a first approach, the concentration of adenosine nucleotides within HCV-infected and non-infected cells was quantified by CE-TOF MS analysis. ATP levels were approximately 7- and 50-fold higher, respectively, than the levels of ADP and AMP in non-infected Huh-7 cells (Figure 1A). At 9 days post-infection with HCV particles produced from a wild-type JFH-1 isolate [14], the intracellular levels of ATP, ADP and AMP were significantly (52–59%) lower than those in naïve Huh-7 cells (Figure 1A). ATP/ADP and ATP/AMP ratios were comparable among HCV-infected and non-infected cells (Figure 1B). A similar result was obtained using JFH-1/4-5 cells that harbor a HCV subgenomic replicon (SGR) RNA derived from the JFH-1 isolate [15]; the intracellular ATP level of JFH-1/4-5 cells was lower than that of parental Huh-7 cells (Figure S1). These findings are basically consistent with a recent report that phosphorylation-mediated activation of AMP-activated protein kinase is inhibited in cells undergoing HCV genome replication, and that ATP/ADP ratios are similar among cells that do and do not demonstrate HCV replication [16], [17].

### Measurement of ATP levels in HCV-replicating cells using ATeam

**Figure 2:**
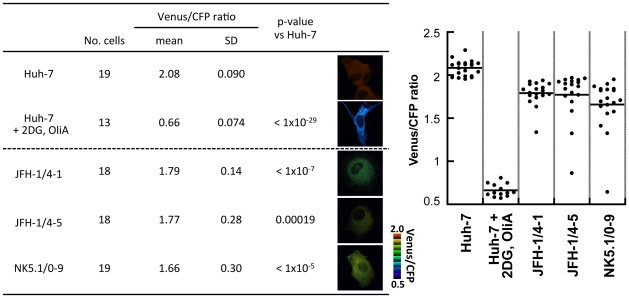
ATP fluctuations within the cytoplasm of HCV replicating cells analyzed using the original ATeam. Huh-7 cells carrying a HCV subgenomic replicon, JFH-1/4-1, JFH-1/4-5 (genotype 2a), and NK5.1/0-9 (genotype 1b) and parental Huh-7 cells were transfected with an ATP probe, AT1.03^YEMK^. Forty-eight hours after transfection, the Venus/CFP emission ratio in the cytoplasm of each cell was calculated from fluorescent images acquired with a confocal microscope FV1000 (Olympus). Huh-7 cells treated with 10 mM 2-DG and 10 µg/ml OliA for 20 min were used as a negative control. Data are presented as means and standard deviation values (SD) for each cell. Statistical differences among Huh-7 cells were evaluated using Student's *t*-test. Pseudocolored images of Venus channel/CFP channel ratios of representative cells and a pseudocolor scale are shown. In the graph on the right, each plot indicates the Venus/CFP ratio of each cell. The horizontal lines in the center represent the mean values for each group.

To visualize ATP levels in living cells undergoing HCV genomic replication, one of the ATeam indicators, AT1.03^YEMK^, which has a high affinity for ATP, was introduced into HCV replicon cells carrying SGR RNA or into parental Huh-7 cells and was imaged using confocal fluorescence microscopy. Consistent with previous observations in HeLa cells [2], this ATP indicator was distributed throughout the cytoplasm. FRET signals (Venus/CFP fluorescence emission ratios), which reflect ATP levels in living cells, were calculated from the fluorescent images of CFP and Venus, a variant of YFP that is resistant to intracellular pH [18], within the cytoplasm of individual cells. Each independent measurement was plotted as indicated in Figure 2. Uniform Venus/CFP ratios were observed in Huh-7 cells. These ratios were reduced dramatically following combined treatment with 2-deoxyglucose (2DG) and Oligomycin A (OliA), which inhibit glycolysis and the oxidative phosphorylation of ADP to ATP, respectively [2]. When AT1.03^YEMK^ was expressed in the HCV replicon-harboring cells JFH-1/4-1, JFH-1/4-5 (genotype 2a) and NK5.1/0-9 (genotype 1b) [15], Venus/CFP ratios were significantly lower than those seen in parental Huh-7 cells. This result is consistent with the mass spectrometry results shown in Figures 1A and S1. Venus/CFP ratios were more variable in the replicon-carrying cells compared to Huh-7 cells. It is possible that ATP levels in the replicon cells correlate with viral replication levels, which may vary among the cells tested.

### The consumption of ATP is increased in HCV-replicating cells

It has been reported that ATP is involved in different steps in the course of HCV replication such as in the initiation of RNA synthesis by NS5B RdRp [9]. NS3 unwinds RNA in an ATP-dependent manner and may be involved in viral replication [11], [19], [20]. NS4A has been shown to enhance the ability of the NS3 helicase to bind RNA in the presence of ATP [21]. In addition, ATP is generally used as a material in RNA synthesis. Together with the above results (Figures 1 and 2), one may hypothesize that active consumption of ATP in cells where HCV RNA replicates efficiently results in lower levels of cytoplasmic ATP compared to cells in the absence of the viral RNA. To study the influence of HCV RNA replication on the consumption of ATP in cells, we used permeabilized HCV replicon cells [13], [22].

**Figure 3:**
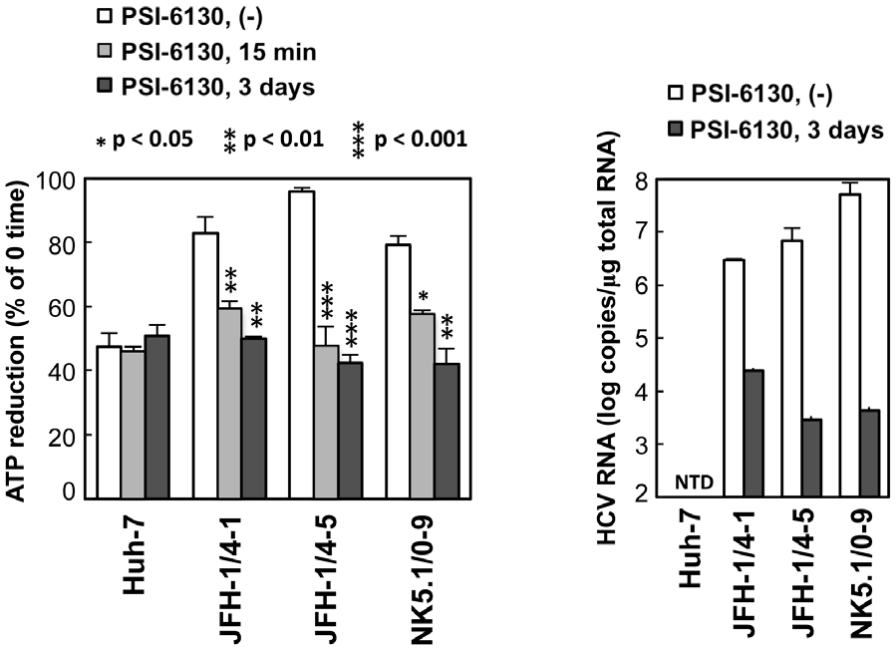
ATP consumption in cells replicating HCV RNA. (Left) The indicated cell lines were pretreated with 10 µM PSI-6130 for 3 days or were cultured in the absence of the drug, followed by trypsinization and permeabilization. ATP-containing reaction buffer plus 10 µM PSI-6130 was added to some of the non-pre-treated cells (PSI-6130, 15 min; light gray bars). ATP-containing PSI-6130-free reaction buffer was added to the rest of the non pre-treated cells (PSI-6130, (−); white bars) and to the pre-treated cells (PSI-6130, 3 days; dark gray bars). After 15 min incubation, ATP levels in cell lysates were measured using a luciferase-based assay. ATP reduction compared to ATP levels at the 0-time point was calculated. The mean values of three independent samples with SD are displayed. Statistical differences between cells treated with and without treatment with PSI-6130 were evaluated using Student's *t*-test. (Right) HCV RNA titers in cells corresponding to the left panel were determined using real-time quantitative RT-PCR. Data are presented as means and SD for three independent samples. NTD indicates not detected.

Following the addition of ATP to permeabilized cells, reduced ATP levels were detected using a luciferase-based assay (see Materials and Methods for details). Fifteen minutes after the addition of ATP, ATP levels in permeabilized replicon-carrying cells (JFH-1/4-1, JFH-1/4-5 and NK5.1/0-9) were reduced by 82–95%, and this reduction was greater than that observed in control Huh-7 cells (47%)(Figure 3). When the replication of HCV RNA was inhibited by pre-treatment of the cells with the cytidine analogue inhibitor of HCV NS5B polymerase, PSI-6130 [23], [24], for 3 days, the reduction in ATP levels in the replicon cells was comparable to that of Huh-7 cells. A decrease in ATP reduction in the replicon cells was observed even following a 15-min treatment with the inhibitor. An effect of inhibition of viral replication on cytoplasmic ATP levels in replicon cells was also observed by ATeam-based analysis of Venus/CFP ratios following inhibition of replication by IFN-alpha (Figure S2). These results suggest that ATP is actively consumed during viral replication in HCV replicon cells, leading to decreased levels of ATP in the cytoplasm.

### Development of a system to monitor ATP levels at putative subcellular sites of HCV replication in single living cells

**Figure 4:**
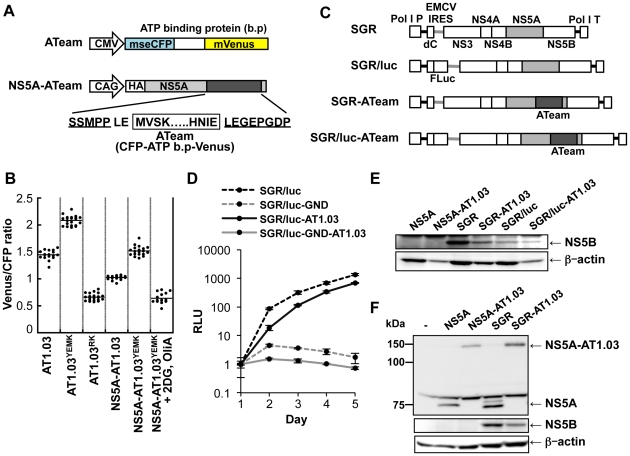
Development of NS5A-ATeam and SGR-ATeam to enable real-time monitoring of ATP. (A) Schematic representation of the ATeam and NS5A-ATeam used in this study. ATeam genes were inserted into the 3′ region of a HA-NS5A expression vector to generate NS5A-ATeam. The underlined sequences indicate NS5A residues. The insertion site was between residues 2394 and 2395, numbered according to the polyprotein of the HCV JFH-1 isolate. CMV, Cytomegalovirus promoter; CAG, CAG promoter; ATP b.p, ATP binding protein. HA, HA tag. (B) Huh-7 cells were transfected with ATeam and NS5A-ATeam constructs. Forty-eight hours post-transfection, the Venus/CFP ratios of each cell were calculated from fluorescent images acquired with a confocal microscope in the same way as described in the legends for Figure 2. Each plot shows the ratio of individual cells. Horizontal lines represent means. (C) Schematic representation of the SGR and SGR-ATeam plasmids used, with or without the firefly luciferase gene (Fluc). HCV polyproteins are indicated by the open boxes. ATeam genes were inserted into the same site in the NS5A C-terminal region. Bold lines indicate the HCV UTR. EMCV IRES is denoted by the gray bars. Pol I P, Pol I promotor; dC, 5′ region of Core gene; Pol I T, Pol I terminator. (D) Replication levels of SGR/luc-AT1.03 in transfected cells were determined by luciferase assay 1–5 days post-transfection. SGR/luc and SGR/luc-GND were used as positive and negative controls, respectively. Values given were normalized for transfection efficiency with luciferase activity determined 24 h post-transfection. All data are presented as means and SD for three independent samples. (E) Huh-7 cells were transfected with constructs encoding NS5A, NS5A-AT1.03, SGR, SGR-AT1.03, SGR/luc or SGR/luc-AT1.03, followed by immunoblotting with anti-NS5B or anti-beta-actin antibody. (F) Cells transfected with constructs encoding NS5A, NS5A-AT1.03, SGR or SGR-AT1.03 were analyzed by immunoblotting with anti-NS5A, anti-NS5B or anti-beta-actin antibodies.

Moradpour et al. have established functional HCV replicons that have either an epitope tag or the coding sequence for a green fluorescent protein (GFP) inserted in frame close to the C-terminus of NS5A, which they used to demonstrate incorporation of the NS5A-GFP fusion protein into the viral RC [25]. To further investigate intracellular changes in ATP during HCV replication, we generated HCV JFH-1-based subgenomic replicons harboring an ATeam insertion in the 3′ region of NS5A (SGR-ATeam), as well as plasmids expressing NS5A-ATeam fusion proteins (NS5A-ATeam)(Figures 4A and 4C).

We first tested whether NS5A-ATeam fusion proteins can be used to monitor ATP levels over a range of concentrations in living cells. The Venus/CFP ratios in individual cells expressing NS5A fused either with AT1.03^YEMK^ (*Kd* = 1.2 mM at 37°C [2]) or with a relatively lower affinity version, AT1.03 (*Kd* = 3.3 mM at 37°C [2]) were measured. As shown in Figure 4B, differences in the Venus/CFP ratios of NS5A- AT1.03^YEMK^ and NS5A-AT1.03 were similar to those of AT1.03^YEMK^ and AT1.03, although average ratios were lower for NS5A- AT1.03^YEMK^ and NS5A-AT1.03 compared to AT1.03^YEMK^ and AT1.03. In the presence of 2DG and OliA, Venus/CFP ratios of NS5A-AT1.03^YEMK^ were markedly reduced to levels that were comparable to those of AT1.03^RK^, an inactive mutant with R122K/R126K substitutions [2]. These results demonstrate that NS5A-ATeams can function as ATP indicators, although their dynamic ranges of Venus/CFP ratios are slightly smaller than those of the original, non-fused ATeams.

We next investigated whether the SGR-ATeam could initiate and sustain transient replication of HCV RNA in cells. A RNA polymerase I (Pol I)-derived plasmid, which carries SGR/luc-AT1.03 containing a luciferase reporter gene ([26]; Figure 4C), or its replication-defective mutant were transfected into Huh-7 cells and levels of viral replication were determined by measuring luciferase activity at various time intervals over a five day period (Figure 4D). Although replication of SGR/luc-AT1.03 was delayed compared with parental SGR/luc, the luciferase activity expressed from SGR/luc-AT1.03 rose to approximately a thousand-fold higher than that expressed from SGR/luc-GND-AT1.03 at five days post-transfection. It appears that SGR-AT1.03, which does not carry the luciferase gene, replicated more efficiently than SGR/luc-AT1.03, as determined by Western blotting of the HCV NS5B protein within cells four days post-transfection (Figure 4E). As indicated in Figure 4F, an abundant protein of the same size as that expected for the NS5A-ATeam fusion protein was observed in cells expressing either NS5A-AT1.03 or SGR-AT1.03, indicating that the NS5A-ATeam fusion protein is stable and is not cleaved during HCV replication. Thus, we concluded that the modified replicon constructs in which the ATeam is incorporated into the NS5A region are functional and remain capable of efficient transient replication of HCV RNA.

### Visualization of ATP levels and distinctive features of ATP distribution in cells replicating ATeam-tagged SGR

**Figure 5:**
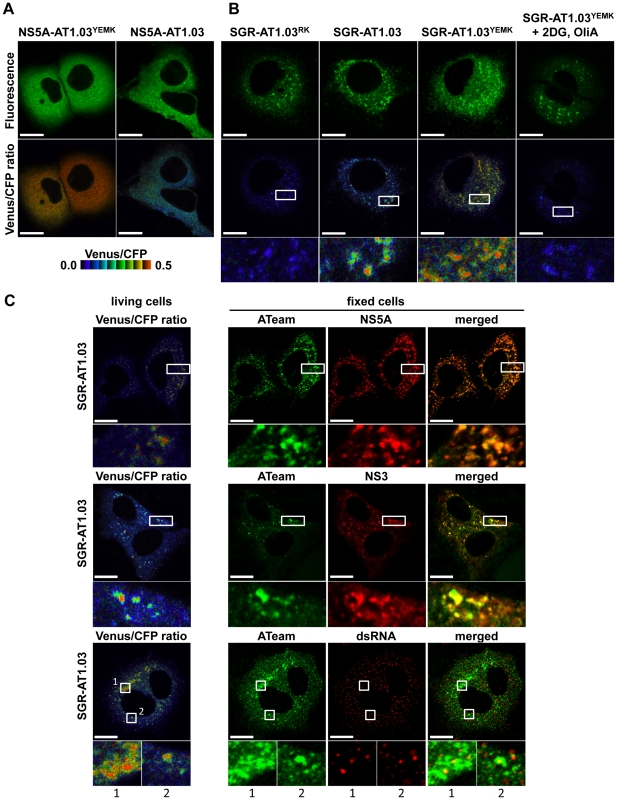
Visualization of sites of focal accumulation of ATP in cells expressing NS5A-ATeam or SGR-ATeam. (A) Huh-7 cells were transfected with NS5A-AT1.03 or NS5A-AT1.03^YEMK^. Four days after transfection, the cells were analyzed using spectral imaging (405-nm excitation) of LSM510-META (Carl Zeiss). Images were processed to the CFP channel (F_CFP_) and the Venus channel (F_Venus_) using a linear unmixing algorithm using a reference for each spectrum. The upper panels demonstrate the signal intensity from a spectral channel with maximum intensity and represent the expression pattern of NS5A-ATeam. The lower panels are constructed from FRET ratio images (F_CFP_/F_Venus_) with pseudocolors. The pseudocolor scale is shown below. Scale bars, 20 µm. (B) Huh-7 cells were transfected with SGR-AT1.03^RK^, SGR-AT1.03 or SGR-AT1.03^YEMK^, and were analyzed in the same way as described in (A). SGR-AT1.03^YEMK^ -transfected cells were treated with 10 mM 2DG and 10 µg/ml OliA just before imaging and were used as a negative control. The upper panels demonstrate the intensity from a spectral channel with maximum intensity and represent the expression pattern of NS5A-ATeam processed from SGR-ATeam. The lower panels indicate square areas within FRET ratio panels magnified five-fold. Scale bars, 20 µm. (C) Cells were fixed after live-cell FRET imaging, and the same cell was analyzed by indirect immunofluorescence staining. Viral proteins were labeled with antibodies against NS5A (upper panels), NS3 (middle panels) and dsRNA (lower panels), which were detected with an Alexa Fluor 555-labeled anti-rabbit or anti-mouse antibody. ATeam panels (green) represent the expression of NS5A-ATeam processed from SGR-ATeam, and NS5A, NS3 or dsRNA panels (red) represent the immunostained signals. Enlarged views of the areas outlined by squares at a five-fold magnification are also shown. Scale bars, 20 µm.

This SGR-ATeam system that was established to analyze cellular ATP levels was used in living HCV RNA-replicating cells in which membrane-associated RCs are formed through the interaction of viral proteins, including NS5A, and cellular proteins. We compared the subcellular distribution of fluorescent signals expressed from NS5A-ATeams and SGR-ATeams using emission-scanning confocal fluorescence microscopy with a Zeiss META detector. NS5A-AT1.03 and NS5A-AT1.03^YEMK^ were diffusely distributed throughout the cytoplasm (Figure 5A; upper panels). Venus/CFP ratios of NS5A-ATeam constructs were almost constant throughout the cytoplasm (Figure 5A; lower). As expected, Venus/CFP ratios in cells expressing NS5A-AT1.03^YEMK^ were markedly higher than those of NS5A-AT1.03 (Figure 5A; lower). In contrast, cells replicating SGR-AT1.03 and SGR-AT1.03^YEMK^ showed foci of brightly fluorescent dot-like structures in the cytoplasm (Figure 5B; upper panels). Interestingly, some of these fluorescent foci had an apparently higher Venus/CFP ratio than the surrounding cytoplasmic region (Figure 5B; middle and lower panels). Although the number of high Venus/CFP ratios was not consistent between the cells, this phenotype was observed in most of the cells that were replicating SGR-AT1.03 (Figure S3). Such high focal Venus/CFP ratios were not detected in cells replicating SGR-AT1.03^RK^ or in SGR-AT1.03^YEMK^ -replicating cells treated with 2DG and OliA. Thus, foci with a high Venus/CFP ratio apparently represent the presence of high ATP levels at distinct sites in cells replicating HCV RNA. In addition, when a replication-defective polyprotein that extended from NS3 through to the NS5B protein, including NS5A-AT1.03, was expressed, no high Venus/CFP ratio was seen in the cells in spite of the fact that NS5A-AT1.03 was detected in dot-like structures throughout the cytoplasm (Figure S4). These results strongly suggest that the high Venus/CFP ratios observed using the SGR-ATeam system are associated with the replication of HCV RNA.

To investigate whether the high Venus/CFP ratios of the dot-like structures detected in cells replicating SGR-ATeam are located at the HCV RC, FRET images of SGR-AT1.03-replicating cells were analyzed, followed immunofluorescence analysis of cells fixed and stained with either anti-NS5A or anti-NS3 antibodies (Figure 5C). Confocal fluorescence microscopy at high magnification demonstrated that the high Venus/CFP ratios that were identified in foci of various sizes were co-localized with NS5A and NS3 that were possibly membrane-bound within the cytoplasm of the viral replicating cells. Some of the NS3- or NS5A-labeled proteins that were identified by immunofluorescence were not associated with high Venus/CFP ratios. These results are consistent with previous reports, which demonstrated that only some of the expressed HCV NS proteins contribute to viral RNA synthesis [27]. To further investigate the relationship between the cellular sites at which there was a high Venus/CFP ratio and HCV RNA replication, double-stranded RNA (dsRNA) was visualized by staining with a specific anti-dsRNA antibody after FRET imaging (Figure 5C). This staining indicated that dsRNA-containing dot-like structures co-localized with structures that displayed high Venus/CFP ratios. Therefore, it is most likely that the dot-like structures with high Venus/CFP ratios that were detected using the SGR-ATeam system reflect the sites of HCV RNA replication or HCV RCs.

Several studies have shown that mitochondria, which play a central role in ATP metabolism, localize to areas near the membranous web, the likely site of HCV RNA replication [28]. We thus compared the subcellular localization of the fluorescence signals detected in cells expressing SGR-ATeam with that of mitochondria that were visualized by staining with Mitotracker. Foci with high Venus/CFP ratios did not colocalize with, but were localized adjacent to mitochondria in cells that were replicating SGR-AT1.03 (Figure S5). This finding might reflect the fact that ATP can be directly supplied from mitochondria to the sites of viral RNA replication in cells.

### Quantification of ATP at putative cytoplasmic sites of HCV RNA replication within cells

**Figure 6:**
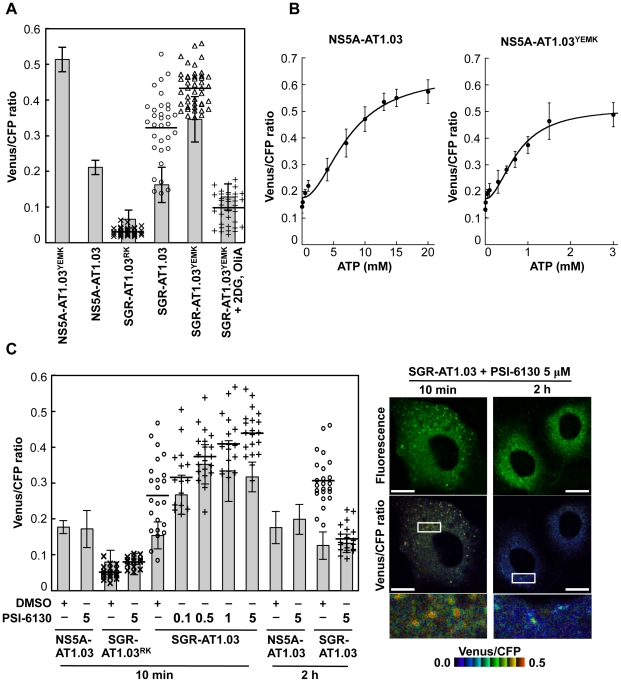
Estimation of ATP levels at possible sites of HCV RNA replication in living cells. (A) Venus/CFP emission ratios were calculated from images of CFP and Venus channels in individual cells for each group. Bar- and dotted graphs indicate ratios within the cytoplasm and ratios for dot-like structures, respectively, in the same cells, as shown in Figures 5A and 5B. Data in bar graphs are indicated as means and SD. Horizontal lines in the dot graphs denote means from at least three independent cells. Values in the cytoplasm of cells transfected with NS5A-AT1.03^YEMK^ and SGR-AT1.03^YEMK^ were statistically significant (p<0.05) as evaluated using the Student's *t*-test. (B) Calibration of NS5A-ATeam in cells under semi-intact conditions. Cells were transfected with NS5A-AT1.03 and NS5A-AT1.03^YEMK^, respectively. Forty-eight hours later, the cells were permeabilized, followed by addition of known concentrations of ATP. FRET analyses were performed as described in Figure 5A. Each trace represents mean with SD of at least six independent cells. Plots were fitted with Hill equations with a fixed Hill coefficient of 2; R = (R_max_−R_min_)×[ATP]^2^/([ATP]^2^+*Kd* ^2^)+R_min_, where R_max_ and R_min_ are the maximum and minimum fluorescence ratios, respectively. *Kd* is the apparent dissociation constant. R values were 0.994 and 0.986 for NS5A-AT1.03 and NS5A-AT1.03^YEMK^, respectively. (C) Cells were transfected with NS5A-AT1.03, SGR-AT1.03^RK^ or SGR-AT1.03. The cells were then treated with PSI-6130 at indicated concentrations (µM) for 10 min or 2 h, and were analyzed as described in (A). Values in the cytoplasm of cells transfected with SGR-AT1.03 with and without PSI-6130 treatment were statistically significant (p<0.05 for control versus 0.1 or 1 µM PSI-6130, p<0.01 for control versus 0.5 or 5 µM PSI-6130) as evaluated using the Student's *t*-test. Representative cells treated with 5 µM PSI-6130 are shown in the right panel. The lower panel is a five-fold magnification of the boxed area. Scale bars, 20 µm.

Based on the above observations, FRET signals detected within cells expressing SGR-ATeam or NS5A-ATeam can be classified as either signals from distinct dot-like structures, which represent putative subcellular sites of HCV RNA replication, or as signals that are diffuse throughout the cytoplasm. The Venus/CFP emission ratio in individual cells into which NS5A-AT1.03, NS5A-AT1.03^YEMK^, SGR-AT1.03, SGR-AT1.03^YEMK^ or SGR-AT1.03^RK^ was introduced was determined (Figure 6A). Fluorescent signals corresponding to cytoplasmic ATP were identified by subtracting signals at putative sites of viral RNA replication from signals from the cytoplasmic area as a whole. Cytoplasmic Venus/CFP ratios within cells replicating SGR-AT1.03 and SGR-AT1.03^YEMK^ were lower than those in cells expressing NS5A-AT1.03 and NS5A-AT1.03^YEMK^, respectively. Therefore, cytoplasmic ATP levels within HCV RNA-replicating cells were lower than in non-replicating cells. This result is consistent with the findings shown in Figure 1A. The average Venus/CFP ratios at potential sites of viral RNA replication were greater than the corresponding cytoplasmic levels in cells replicating SGR-AT1.03 or SGR-AT1.03^YEMK^. As expected, a significant decrease in Venus/CFP ratios was observed in cells treated with 2DG and OliA.

We next quantified ATP levels within individual cells replicating HCV RNA based on the Venus/CFP ratios obtained. To generate standard curves for this calculation, permeabilized cells expressing NS5A-AT1.03 or NS5A-AT1.03^YEMK^ were prepared by digitonin treatment, followed by the addition of defined concentrations of ATP and subsequent FRET analysis [29], [30]. As shown in Figure 6B, under these experimental conditions, baseline Venus/CFP ratios of approximately 0.1 were detected in the absence of exogenous ATP, and Venus/CFP ratios were observed to increase linearly with increasing ATP concentration. The standard curves thus obtained can be used to estimate the ATP concentrations of unknown samples in which a particular ATeam containing an ATP probe at the C terminus of HCV NS5A, such as NS5A-ATeam or SGR-ATeam, have been introduced. Based on the fluorescent signal obtained in cells replicating SGR-ATeam, as well as in cells expressing NS5A-ATeam, the ATP concentration at putative sites of HCV RNA replication was estimated to be ∼5 mM in the experiments shown in Figures 5A and 5B (average value of putative replication sites; 4.8 mM). After subtraction of the ATP that was localized at the HCV replication sites, the ATP concentration of HCV-replicating SGR cells (∼1 mM) was found to be approximately half that observed in parental non-replicating cells (∼2 mM)(average values in SGR and parental cells; 0.8 mM and 2.2 mM, respectively). To our knowledge, this is the first experiment in which ATP levels were estimated inside living cells during viral genome replication.

Figures 5 and 6A demonstrate changes in ATP concentrations at distinct sites in cells undergoing HCV RNA replication. Finally, we determined the effect of the PSI-6130 inhibitor of HCV replication on the change in subcellular ATP concentration in cells following introduction of SGR-AT1.03, SGR-AT1.03^RK^ or NS5A-AT1.03 (Figure 6C). In general, nucleoside analogue inhibitors of viral replication prevent RNA/DNA synthesis by chain termination immediately after addition to infected cells [23]. Indeed, as shown in Figure 3, a decrease in ATP consumption was detected even following a PSI-6130 treatment period as short as 15 min of permeabilized HCV replicon cells. We therefore analyzed and estimated ATP levels in cells in the presence of PSI-6130 for 10 min and 2 h. ATP concentrations at putative sites of viral RNA replication, as well as cytoplasmic ATP levels, were higher in SGR-AT1.03-replicating cells in the presence of 0.1–5 µM PSI-6130 for 10 min compared to the same cells without inhibitor treatment or to NS5A-AT1.03-expressing cells. A dose-dependent PSI-6130-induced increase in ATP levels at the putative replication sites was observed under the condition used. By treatment with PSI-6130 for 2 h, the ATP levels at putative replication sites were significantly lower than those without inhibitor treatment in SGR-AT1.03-replicating cells. The cytoplasmic ATP levels were similar with or without 2-h treatment (Figure 6C). In HCV SGR-ATeam cells treated with PSI-6130 for 3 days, HCV RNA replication was dramatically inhibited by greater than 90% with no observed cytotoxicity (Figure S6) and, as expected, little or no high Venus/CFP signal was detected anywhere in the cells (data not shown). We adapted the ATeam system to monitor ATP in HCV RNA replicating cells and found increased ATP levels at the putative subcellular sites of the viral replication. Findings obtained from experiments using the viral polymerase inhibitor strongly suggest that changes in ATP concentrations at the distinct sites observed depend on the viral RNA replication.

## Discussion

This paper is the first to demonstrate changes in ATP within cells during viral genome replication. ATP requirements during the virus lifecycle have been studied for years. Several key steps during the viral life cycle, such as genome synthesis, require high-energy phosphoryl groups. For instance, it has been shown that ATP is required for the formation of a preinitiation complex for de novo RNA synthesis by RdRp of flaviviruses [31]. Transcriptional initiation and RNA replication by influenza virus RdRp are functional in an ATP-dependent fashion [32], [33]. An ATP requirement of viral helicase activities has also been reported [34]. Furthermore, it has been demonstrated that ATP is involved in the assembly and/or release of viral structural proteins possibly via interaction with ATP-dependent chaperones [35], [36]. However, it has been controversial as to whether ATP can be concentrated in particular subcellular compartment(s) in infected cells during viral replication. One of the underlying reasons for this controversy may be that a method by which cellular ATP levels can be determined, apart from examination of ATP levels in cellular extracts in the steady-state, has been lacking [37]. Recently Imamura et al. established FRET-based indicators, known as ATeams, for ATP quantification, and have shown that the use of ATeams enables the monitoring of ATP levels in real-time in different cellular compartments within individual cells [2].

In this study, in order to visualize and monitor ATP levels in living cells during replication of the viral genome, we first introduced the original ATeam-expressing plasmids into cells and found that cytoplasmic ATP levels in cells undergoing HCV genotype 1b and 2a RNA replication were lower than those in cured or parental cell lines (Figures 2 and S2). These results agree with the results of CE-TOF MS analysis (Figure 1) and the ATP consumption assay (Figure 3). It is therefore likely that ATP is actively consumed in cells during viral RNA replication, resulting in reduced levels of ATP in the cytoplasm. Furthermore, NS5A-ATeam fusion constructs, in which the ATeam gene was introduced into the C-terminal end of the NS5A coding region, and SGR-ATeam constructs containing a HCV JFH-1-derived subgenomic replicon within the NS5A-ATeam fused sequence as described above, were engineered (Figure 4). The results obtained using several ATeam fusion constructs with different affinities for ATP indicated that NS5A-ATeam fusion constructs can be used as FRET-based ATP indicators, and that the ATeam-tagged HCV replicons are capable of transient replication of viral RNA (Figure 4). It is interesting that our experiment using a SGR-ATeam construct provides evidence for the formation of ATP-enriched foci within cells that support HCV RNA replication (Figures 5 and 6). FRET-signal detection followed by indirect immunofluorescence allowed us to visualize co-localization of viral proteins as well as dsRNA at sites of ATP accumulation in cells (Figure 5), suggesting that these membrane-associated ATP-enriched foci likely represent sites of HCV RNA replication in transient replication assays.

Attempting to precisely quantify ATP within individual cells or particular intracellular compartments is a very challenging process. The luciferin-luciferase reaction has been utilized to monitor cellular ATP levels by measuring the released photon count during catalysis of bioluminescent oxidation by firefly luciferase. A previous study based on the luciferin-luciferase assay estimated basal cytoplasmic ATP levels at ∼1.3 mM, which increased to ∼5 mM during apoptotic cell death [38]. However, the results obtained were likely influenced by cellular levels of luciferase and other assay components, as well as by the pH of the cells. In this study, we describe quantification of ATP in human hepatoma Huh-7 cells undergoing HCV RNA replication using SGR-ATeam technology. Although ATP requirements during the lifecycles of various viruses have been studied for years, the use of ATeam technology enabled us, for the first time, to evaluate ATP concentrations at sites of viral replication within living cells. We here demonstrate that ATP concentrations at these putative subcellular sites of HCV RNA replication approach ∼5 mM (Figure 6). This ATP level is as high as that observed during apoptotic processes such as caspase activation and DNA fragmentation, even though the latter ATP level was determined using a different assay system [38]. Considering that these apoptotic events were not observed at basal ATP levels [38], replication of the viral genome likely also requires high concentrations of cellular ATP. It should be noted that, in contrast to the fluorescent reporter system traditionally used to calculate the ATP/ADP ratio [39], the bacterial epsilon subunit used in ATeam is highly specific for ATP, but not for other nucleotides such as ADP, CTP, GTP or UTP [2], [3]. In evaluating the effect of the HCV polymerase inhibitor on changes in the subcellular ATP concentration in cells replicating SGR-ATeam, an increase in ATP concentration was observed both at putative replication sites and in the cytoplasm of SGR-AT1.03-replicating cells in the presence of PSI-6130 for 10 min (Figure 6C). By contrast, 2-h treatment with the inhibitor resulted in reduction of ATP levels at putative replication sites in the replicon cells. Although the result of the experiment with 10-min treatment may be somewhat unexpected, it might possibly be explained by the following hypothesis. PSI-6130 began to inhibit viral RNA synthesis, leading to a decrease in ATP consumption. Since a mechanism for ATP transport mediated by host cell and/or viral factor(s) is still active during this time period, the ATP level at the replication sites should be increased compared to that during active replication. Higher levels of metabolic intermediates for glyconeogenesis as well as for glycolysis in HCV-infected cells compared to non-infected cells as determined via metabolome analysis (data not shown) may also be implicated in the increased ATP levels at the initial stage of inhibition of HCV replication. It is likely that active consumption of ATP caused by HCV replication and ATP transportation into the replication sites would lead to reduction of cytoplasmic ATP level. Such a change in ATP balance may result in induction of ATP generation and increase in certain metabolic intermediates related to glucose metabolism. These metabolome responses are supposed to maintain in short-term (10 min) treatment with PSI-6130. Thus, inhibition of HCV RNA replication by PSI-6130 under the conditions used may lead to increase in the cytoplasmic ATP level. It is likely that these metabolome responses were not observed after the longer-term (2 h) treatment presumably because the viral replication was inhibited by the inhibitor for a sufficient period of time. Further study is required to address the molecular mechanism underlying change in ATP balance caused by HCV replication and the viral inhibitors.

The mechanism by which ATP accumulates at potential sites of HCV RNA replication remains unclear. We have previously demonstrated that creatine kinase B (CKB), which is an ATP-generating enzyme and maintains cellular energy stores, accumulates in the HCV RC-rich fraction of viral replicating cells [22]. Our earlier results suggest that CKB can be directed to the HCV RC via its interaction with the HCV NS4A protein and thereby functions as a positive regulator for the viral replicase by providing ATP [22]. One may hypothesize that recruitment of the ATP generating machinery into the membrane-associated site, through its interaction with viral proteins comprising the RC, is at least in part linked with elevated concentrations of ATP at a particular site. Through our preliminary study, however, subcellular ATP distribution was not changed significantly in replicon cells where HCV RNA replication was reduced ∼50% by siRNA-mediated knockdown of the CKB gene (data not shown). Another possibility may be implication of communication between mitochondria and membrane-enclosed structures of HCV RC in ATP transport through membrane-to-membrane contact. As indicated in Figure S5, putative sites of the viral RNA replication with high Venus/CFP ratios were mainly localized proximal to mitochondria. Studies are ongoing to understand the mechanism(s) underlying this phenomenon, as well as to determine if changes in ATP levels at intracellular sites supporting replication might also be observed for other RNA or DNA viruses.

In summary, we have used a FRET-based ATP indicator called ATeam to monitor ATP levels in living cells where viral RNA replicates by designing HCV replicons harboring wild-type or mutated ATeam probes inserted into the C-terminal domain of NS5A. We evaluated changes in ATP levels during HCV RNA replication and demonstrated elevated ATP levels at putative sites of replication following detection of FRET signals, which appeared as dot-like foci within the cytoplasm. The ATeam system may become a powerful tool in microbiology research by enabling determination of subcellular ATP localization in living cells infected or associated with microbes, as well as investigation of the regulation of ATP-dependent processes during the lifecycle of various pathogens.

## Materials and Methods

### Chemicals

PSI-6130 (β-_D_-2′-Deoxy-2′-fluoro-2′-*C*-methylcytidine) and recombinant human IFN-alpha2b were obtained from Pharmasset Inc. (Princeton, NJ) [23], [24] and Schering-Plough (Kenilworth, NJ), respectively. OliA and 2DG were purchased from Sigma-Aldrich (St. Louis, MO). ATP used in this study was complexed with equimolar concentrations of magnesium chloride before use in the experiments.

### Plasmids

The construction of the ATeam plasmids pRSET-AT1.03, pRSET-AT1.03^YEMK^ and pRSET-AT1.03^R122K/R126K^, which express wild-type ATeam (AT1.03), as well as a high-affinity mutant (AT1.03^YEMK^) and a non-binding mutant (AT1.03^RK^), has been previously described [2]. pHH/SGR-Luc (also termed SGR/luc) contains cDNA of a subgenomic replicon of HCV JFH-1 isolate (genotype 2a; [14]) with firefly luciferase flanked by the Pol I promoter and the Pol I terminator, yielding efficient RNA replication upon DNA transfection [26]. pHH/SGR-Luc/GND (also termed SGR/luc-GND), in which a point mutation of the GDD motif of the NS5B was introduced in order to abolish RNA-dependent RNA polymerase activity, was used as a negative control. pHH/SGR (also termed SGR) was created by deleting the luciferase gene in pHH/SGR-Luc. To generate a series of SGR-ATeam plasmids, wild-type or mutant ATeam genes were inserted into pHH/SGR-Luc or pHH/SGR at the Xho I site of NS5A (between amino acids 418 and 419) [25]. The ATeam genes were also inserted into the same site of pCAGNS5A, which contains the NS5A gene of JFH-1 downstream of the CAG promoter and hemagglutinin (HA) tag [26], yielding NS5A-ATeam plasmids. To generate a plasmid expressing NS3-NS5B-AT1.03 under the control of the CAG promoter, a DNA fragment containing the coding region of NS3/NS4A/NS4B/NS5A-AT1.03/NS5B of SGR/luc-ATeam was inserted into the pCAGGS vector [40]. Exact cloning strategies are available upon request.

### Cell culture and plasmid transfection

Human hepatoma Huh-7 cells were propagated in Dulbecco's modified Eagle's medium (DMEM) supplemented with 10% fetal calf serum (FCS) as well as minimal essential medium non-essential amino acid (MEM NEAA)(Invitrogen, Carlsbad, CA) in the presence of 100 units/ml of penicillin and 100 µg/ml of streptomycin. The Huh-7-derived cell lines JFH-1/4-1 and JFH-1/4-5, which support replication of SGR RNA of HCV JFH-1 (genotype 2a) and NK5.1/0-9, which carries the SGR RNA of Con1 NK5.1 (genotype 1b), were cultured and maintained under previously described conditions [15]. DNA transfection was performed using a TransIT-LT1 transfection reagent (Takara, Shiga, Japan) in accordance with the manufacturer's instructions.

### CE-TOF MS analysis

Huh-7 cells were mock-infected or infected with HCVcc derived from a wild-type JFH-1 isolate at a multiplicity of infection of 1. When most cells had become virus positive, as confirmed by immunofluorescence, with no observable cell damage at 9 days post-infection, equal amounts of cells with and without HCV infection were scraped with MeOH including 10 µM of an internal standard after washing twice with 5% mannitol solution. Replicon cells (JFH-1/4-5) that were cultured in the absence of G418 for 2 days were harvested and prepared as above. The extracts were mixed with chloroform and water, followed by centrifugation at 2,300× *g* for 5 min at 4°C, The upper aqueous layer was centrifugally filtered through a 5-kDa cutoff filter to remove proteins. The filtrate was lyophilized and dissolved in water, then subjected to CE-TOF MS analysis. CE-TOF MS experiments were performed using an Agilent CE-TOF MS system (Agilent Technologies, Waldbronn, Germany) as described previously [41].

### ATP consumption assay

The ATP consumption assay using permeabilized replicon cells was carried out as previously described [13], [22] with slight modifications, so that it was unnecessary to add either exogenous phosphocreatine or creatine phosphokinase to minimize ATP reproduction in cells. Cells (2×10^6^) cultured in the presence or absence of PSI-6130 for 72 h were treated with 5 µg Actinomycin D/ml, followed by trypsinization and 3 washes with cold buffer B (20 mM HEPES-KOH [pH 7.7], 110 mM potassium acetate, 2 mM magnesium acetate, 1 mM EGTA, and 2 mM dithiothreitol). The cells were permeabilized by incubation with buffer B containing 50 µg/ml digitonin for 5 min on ice and the reaction was stopped by washing 3 times with cold buffer B. The permeabilized cells (1×10^5^) were resuspended with 100 µl buffer B containing 5 µM ATP, GTP, CTP, and UTP, 20 µM MgCl_2_, and 5 µg/ml Actinomycin D. After incubation at 27°C for 15 min, samples were centrifuged, and 20 µl of the supernatant was then mixed with 5 µl of 5× passive lysis buffer (Promega, Madison, WI). The ATP level was determined using a CellTiter-Glo Luminescent cell viability assay system (Promega). All assays were performed at least in triplicate.

### Live cell microscopy

Plasmids carrying the ATP indicators were transfected at 48 h (ATeam and NS5A-ATeam) or 4 days (SGR-ATeam) before imaging of the cells. One day before imaging, the cells were seeded onto 30-mm glass-bottomed dishes (AGC Techno Glass, Chiba, Japan) at about 60% confluency. For imaging, the cells were maintained in phenol red-free DMEM containing 20 mM HEPES-KOH [pH 7.7], 10% FCS and MEM NEAA.

Two kinds of confocal microscopies were used to perform the FRET analysis in this study as follows. Since the ways of acquisition of each spectrum were quite different between the two microscopies, differences in the values of the Venus/CFP ratios in different experiments were observed. In Figures 2, 4B and S2, cells were imaged using a confocal inverted microscope FV1000 (Olympus, Tokyo, Japan) equipped with an oil-immersion 60× Olympus UPlanSApo objective (NA = 1.35). Cells were maintained on the microscope at 37°C with a stage-top incubation system (Tokai Hit, Shizuoka, Japan). Cells were excited by a 405-nm laser diode, and CFP and Venus were detected at 480–500 nm and 515–615 nm wavelength ranges, respectively. In the analysis shown in Figures 5, 6, S3, S4 and S5, FRET images were obtained using a Zeiss LSM510 Meta confocal microscope with an oil-immersion 63× Zeiss Plan-APOCHROMAT objective (NA = 1.4)(Carl Zeiss, Jena, Germany). Cells were maintained on the microscope at 37°C with a continuous supply of a 95% air and 5% CO_2_ mixture using a XL-3 incubator (Carl Zeiss). Cells were excited by a 405-nm blue diode laser, and emission spectra of 433–604 nm wavelength range were obtained using an equipped scanning module (META detector) [42], [43]. Images were computationally processed by a linear unmixing algorithm using the reference spectrum of CFP and Venus, which were obtained from individual fluorescence-expressing cells. All image analyses were performed using MetaMorph (Molecular Devices, Sunnyvale, CA). Fluorescence intensities of cytoplasmic areas in NS5A-ATeam transfected cells were calculated by subtraction of the signal intensities of the nucleus from the signal intensities of the whole cell, which was standardized by the area of the corresponding cytoplasmic region. Fluorescence intensities of cytoplasmic areas and at dot-like structures corresponding to the putative viral replicating sites in SGR-ATeam-transfected cells were measured and calculated as follows. All pixels above CFP intensity levels of 100–200 were selected. The positions of dot-like structures were then determined by examining areas greater than 0.5×10^−12^ square meters and the intensity of each dot was measured. The fluorescence intensity of the cytoplasmic area, excluding that of the putative viral replicating sites in each cell, was calculated by subtraction of the signal intensities of the nucleus and the dot-like structures, as determined above, from the signal intensity of the whole cell, which was standardized by the area of the corresponding cytoplasmic region. Each Venus/CFP emission ratio was calculated by dividing pixel-by-pixel a Venus image with a CFP image.

To investigate the relationship between Venus/CFP ratios and ATP concentrations in cells, calibration procedures were performed according to previous reports [29], [30]. Huh-7 cells were transfected with NS5A-AT1.03 or NS5A-AT1.03^YEMK^. Forty-eight hours later, the cells were permeabilized by incubation with buffer B containing 50 µg/ml digitonin for 5 min at room temperature. The reaction was stopped by washing 3 times with buffer B, followed by the addition of known concentrations of ATP in warmed medium for imaging. FRET analysis, with calibration of the signal intensity in the cytoplasm of each cell, was performed as described above. Plots were fitted with Hill equations with a fixed Hill coefficient of 2; R = (R_max_−R_min_)×[ATP]^2^/([ATP]^2^+*Kd* ^2^)+R_min_, where R_max_ and R_min_ are the maximum and minimum fluorescence ratios, respectively and *Kd* is the apparent dissociation constant.

To analyze the effect of an inhibitor against HCV NS5B polymerase, the medium for the cells replicating SGR-ATeam was changed to medium containing various concentrations of PSI-6130. After 10-min incubation at 37°C under a continuous supply of 95% air and 5% CO_2_, fluorescence intensities of cytoplasmic areas and at dot-like structures were determined as described above. Medium containing 0.01% DMSO was used as a negative control.

To visualize mitochondria, MitoTracker Red CMXRos (Molecular Probes, Eugene, OR) was added to the culture medium to a final concentration of 100 nM, incubated for 15 min at 37°C and the cells were then washed twice with phosphate buffered saline (PBS) before FRET analysis of living cells. Images were computationally processed as described above. The reference spectrum of MitoTracker Red CMXRos was obtained from stained parental, non-transfected, Huh-7 cells.

### Indirect immunofluorescence

Cells expressing SGR-ATeam were cultured in 30-mm glass-bottomed dishes with an address grid on the coverslip (AGC Techno Glass). After FRET analysis of living cells as described above, the cells were fixed with 4% paraformaldehyde at room temperature for 30 min. After washing with PBS, the cells were permeabilized with PBS containing 0.3% Triton X-100 and individually stained with a rabbit polyclonal antibody against NS3 [44], an anti-NS5A antibody [45], or a mouse monoclonal antibody against dsRNA antibody (Biocenter Ltd., Szirak, Hungary) [46]. The fluorescent secondary antibody used was Alexa Fluor 555-conjugated anti-rabbit- or anti-mouse IgG (Invitrogen). The cells were imaged using a Zeiss LSM510 Meta confocal microscope with an oil-immersion 63× Zeiss Plan-APOCHROMAT objective (NA = 1.4). For dual-color imaging, the ATeam signal was excited with the 488-nm laser line of an argon laser and Alexa Fluor 555 was excited with a 543-nm HeNe laser under MultiTrack mode. Emission filters with a 505- to 530-nm band-pass and 560-nm-long pass filter were used.

### Luciferase assay

Huh-7 cells transfected with SGR/luc or SGR/luc-ATeam were harvested at different time points after transfection (Figure 4D) or at 3 days after treatment with PSI-6130 (Figure S6) and lysed in passive lysis buffer (Promega). To monitor HCV RNA replication, the luciferase activity in cells was determined using a Luciferase Assay system (Promega). All assays were performed at least in triplicate.

### MTT assay

Cell viability was assessed using the Cell Proliferation Kit II (Roche, Indianapolis, IN) according to the manufacturer's instructions. The kit measures mitochondrial dehydrogenase activity, which is used as a marker of viable cells, using a colorimetric sodium3′-[1(-phenylaminocarbonyl)-3,4-tetrazolium]-bis(4-methoxy-6-nitro)benzene sulfonic acid hydrate (MTT) assay.

### Quantification of HCV RNA

HCV RNA copies in the replicon cells with or without PSI-6130 treatment were determined using the real-time detection reverse transcription polymerase chain reaction (RTD-PCR) described previously [47] with the ABI Prisom 7700 sequence detector system (Applied Biosystems Japan, Tokyo, Japan).

### Western blotting

The proteins were transferred onto a polyvinylidene difluoride membrane (Immobilon; Millipore, Bedford, MA) after separation by SDS-PAGE. After blocking, the membranes were probed with a rabbit polyclonal anti-NS5A antibody [44], a rabbit polyclonal anti-NS5B antibody (Chemicon, Temecula, CA), or a mouse polyclonal anti-beta-actin antibody (Sigma-Aldrich), followed by incubation with a peroxidase-conjugated secondary antibody and visualization with an ECL Plus Western blotting detection system (GE Healthcare, Buckinghamshire, UK).

## Supporting Information

Figure S1 **ATP Levels in HCV replicon cells and parental Huh-7 cells determined by CE-TOF MS.** ATP metabolites in Huh-7 cells and JFH-1/4-5 cells were measured by CE-TOFMS. The values of each measurement are shown at left. The right graph shows means with SD of the data at left. Open bar; Huh-7 cells, gray bar; JFH-1/4-5 cells.
(TIF)

Figure S2 **Cytoplasmic ATP levels in HCV replicon cells and IFN-treated cells.** (Left) The HCV replicon cells JFH-1/4-1, JFH-1/4-5 (genotype 2a) and NK5.1/0-9 (genotype 1b), and parental Huh-7 cells were cultured for 72 h in the absence or presence of 1,000 IU/ml IFN-alpha. Forty-eight hours after transfection with AT1.03, the Venus/CFP emission ratio of each cell was calculated from fluorescent images acquired with the confocal microscope FV1000. All data are presented as means and SD for at least 10 independent cells. (Right) HCV RNA titers in cells corresponding to the left panel were determined using real-time quantitative RT-PCR. Data are presented as means and SD for three independent samples. NTD indicates not detected.
(TIF)

Figure S3 **Increase in ATP-enriched dot-like structures in cells replicating SGR-ATeam.** Huh-7 cells were transfected with SGR-AT1.03, and analyzed in the same way as described in the legends for Figures 5A and 5B. The lower four panels are five-fold magnifications of the boxed areas in independent cells. Scale bars, 40 µm.
(TIF)

Figure S4 **Visualization of the ATP level in cells expressing replication-defective HCV polyprotein.** (A) A schematic representation of the NS3-NS5B-AT1.03 plasmid is shown. The HCV polyprotein is indicated by the open boxes. The ATeam gene was inserted into the same site as that for NS5A-ATeam and SGR-ATeam insertion as indicated in the legend for Figure 4A. CAG, CAG promoter. (B) Cells transfected with constructs encoding NS5A, NS5A-AT1.03, NS3-NS5B-AT1.03, SGR or SGR-AT1.03 were analyzed by immunoblotting with anti-NS5A, anti-NS5B or anti-beta-actin antibodies. (C) Huh-7 cells were transfected with NS3-NS5B-AT1.03, and analyzed in the same way as described in the legends for Figures 5A and 5B. The upper panel (Fluorescence) demonstrates signal intensity from a spectral channel with maximum intensity and represents the expression pattern of NS5A-ATeam processed from NS3-NS5B-AT1.03. The lower panels (Venus/CFP ratio) indicate the FRET ratio and a five-fold magnification of the boxed area. Scale bar, 20 µm.
(TIF)

Figure S5 **Relationship between ATP-enriched dot-like structures and mitochondria.** Huh-7 cells replicating SGR-AT1.03 (right panels) and parental cells (left panel) were analyzed. Active mitochondria were labeled with MitoTracker Red CMXRos in living cells, and were analyzed in the same way as described in the legends for Figures 5A and 5B, using a reference for the MitoTracker spectrum. The lowest panels of SGR-ATeam cells indicate five-fold magnifications of the boxed areas. Scale bars, 20 µm.
(TIF)

Figure S6 **Inhibitory effect of PSI-6130 on HCV RNA replication.** (A) Replication levels of SGR/luc-AT1.03 RNA in transfected cells were determined by luciferase assay 3 days after treatment with PSI-6130 at the indicated concentrations (µM). The values shown were normalized for transfection efficiency with luciferase activity determined 24 h post-transfection. All data are presented as means and SD for three independent samples. (B) Cell viability was assessed using the MTT assay.
(TIF)
