# Supplementary figures and images for: Visualization and Measurement of ATP Levels in Living Cells Replicating Hepatitis C Virus Genome RNA
Source: PLoS Pathog. 2012 Mar 1;8(3):e1002561. doi: 10.1371/journal.ppat.1002561 (PMC3291659; doi:10.1371/journal.ppat.1002561)

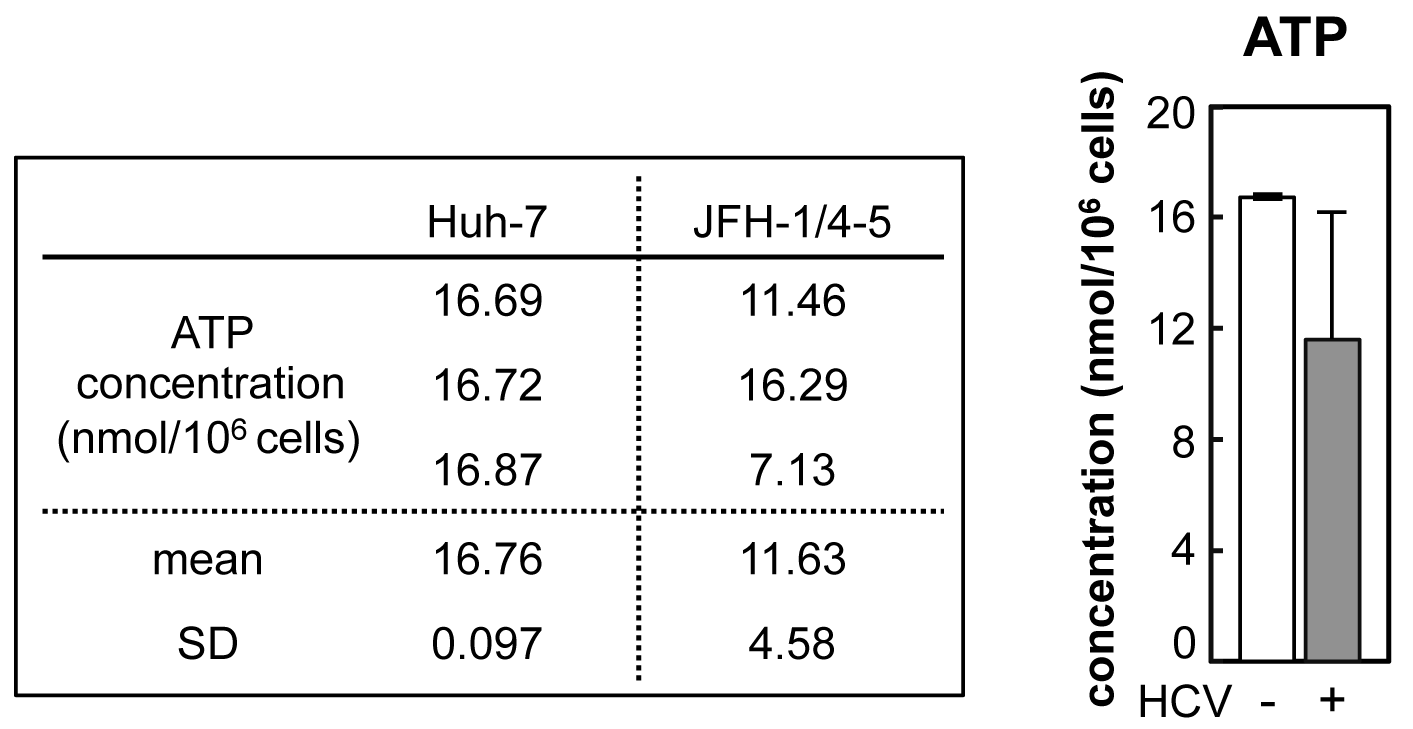

Supplement: Figure S1 — ATP Levels in HCV replicon cells and parental Huh-7 cells determined by CE-TOF MS. ATP metabolites in Huh-7 cells and JFH-1/4-5 cells were measured by CE-TOFMS. The values of each measurement are shown at left. The right graph shows means with SD of the data at left. Open bar; Huh-7 cells, gray bar; JFH-1/4-5 cells. (TIF) [file ppat.1002561.s001.tif]

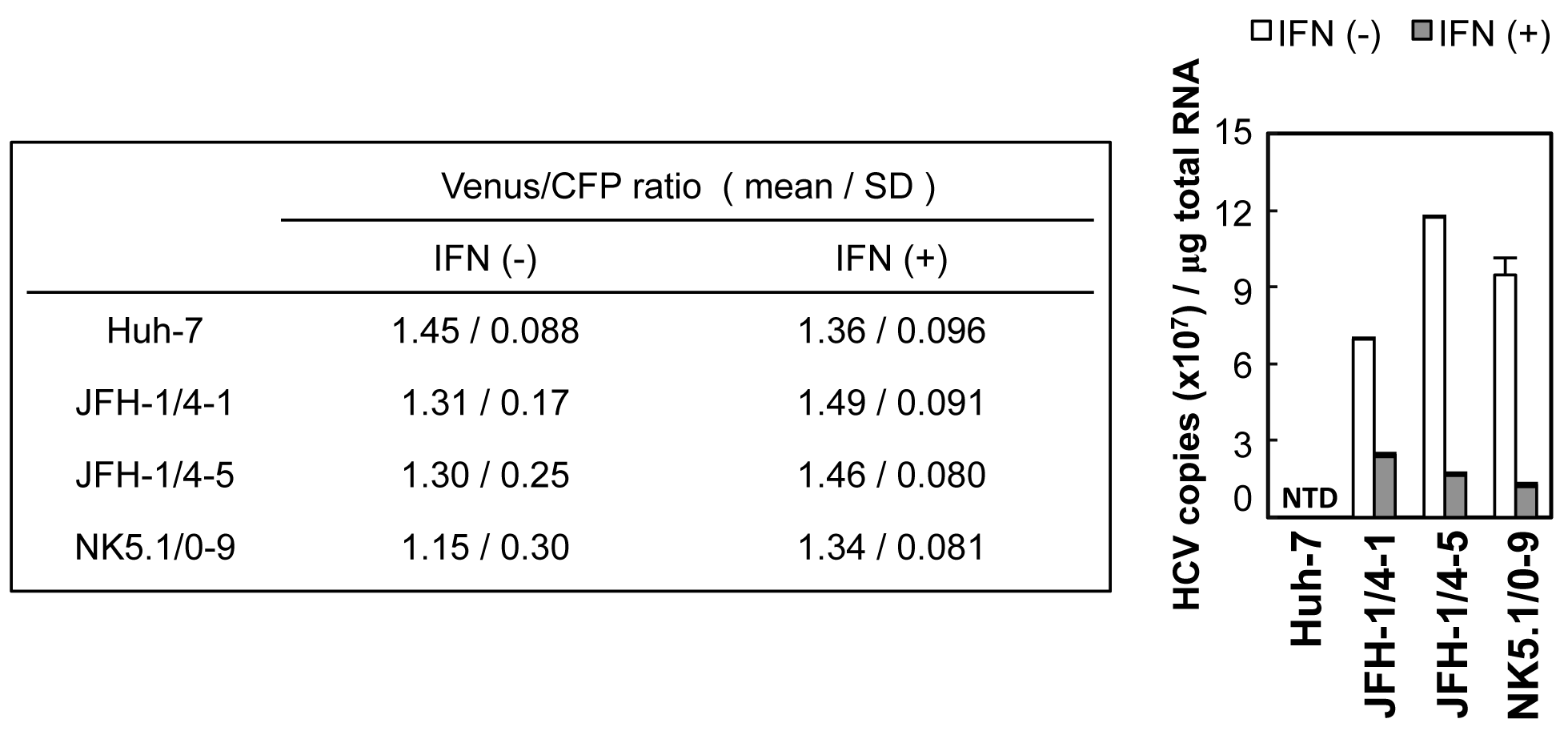

Supplement: Figure S2 — Cytoplasmic ATP levels in HCV replicon cells and IFN-treated cells. (Left) The HCV replicon cells JFH-1/4-1, JFH-1/4-5 (genotype 2a) and NK5.1/0-9 (genotype 1b), and parental Huh-7 cells were cultured for 72 h in the absence or presence of 1,000 IU/ml IFN-alpha. Forty-eight hours after transfection with AT1.03, the Venus/CFP emission ratio of each cell was calculated from fluorescent images acquired with the confocal microscope FV1000. All data are presented as means and SD for at least 10 independent cells. (Right) HCV RNA titers in cells corresponding to the left panel were determined using real-time quantitative RT-PCR. Data are presented as means and SD for three independent samples. NTD indicates not detected. (TIF) [file ppat.1002561.s002.tif]

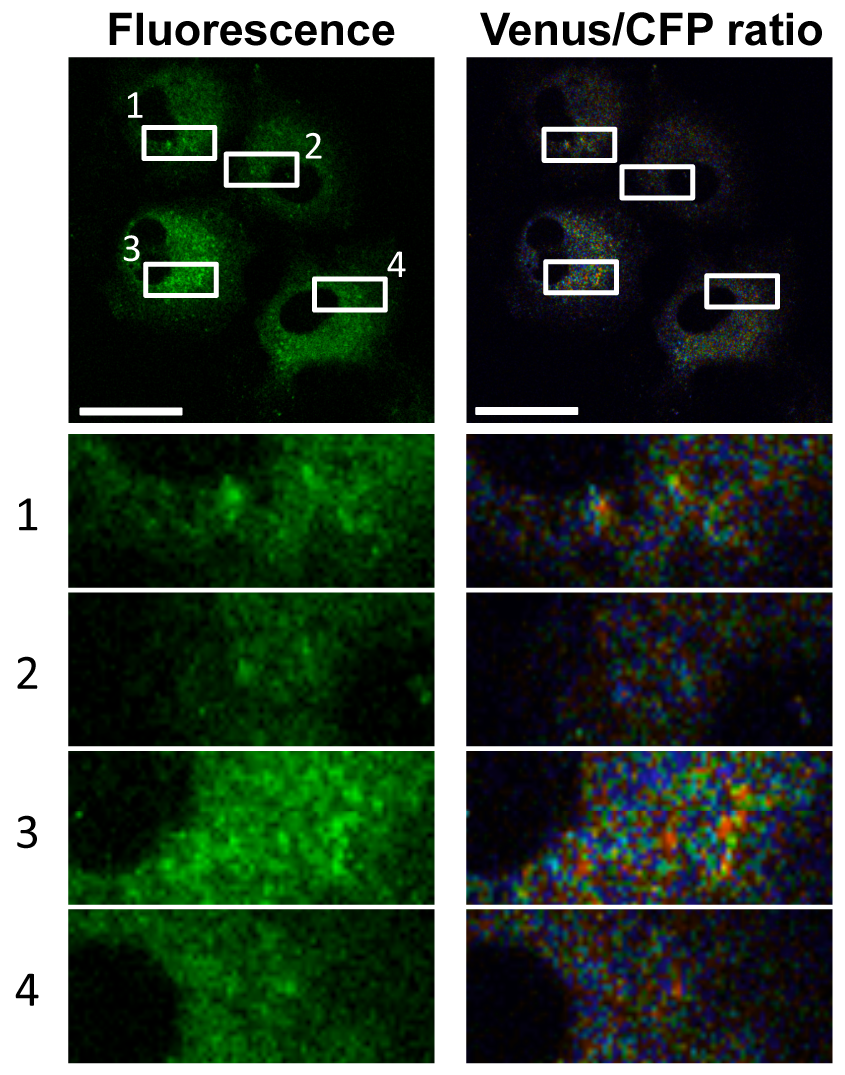

Supplement: Figure S3 — Increase in ATP-enriched dot-like structures in cells replicating SGR-ATeam. Huh-7 cells were transfected with SGR-AT1.03, and analyzed in the same way as described in the legends for Figures 5A and 5B. The lower four panels are five-fold magnifications of the boxed areas in independent cells. Scale bars, 40 µm. (TIF) [file ppat.1002561.s003.tif]

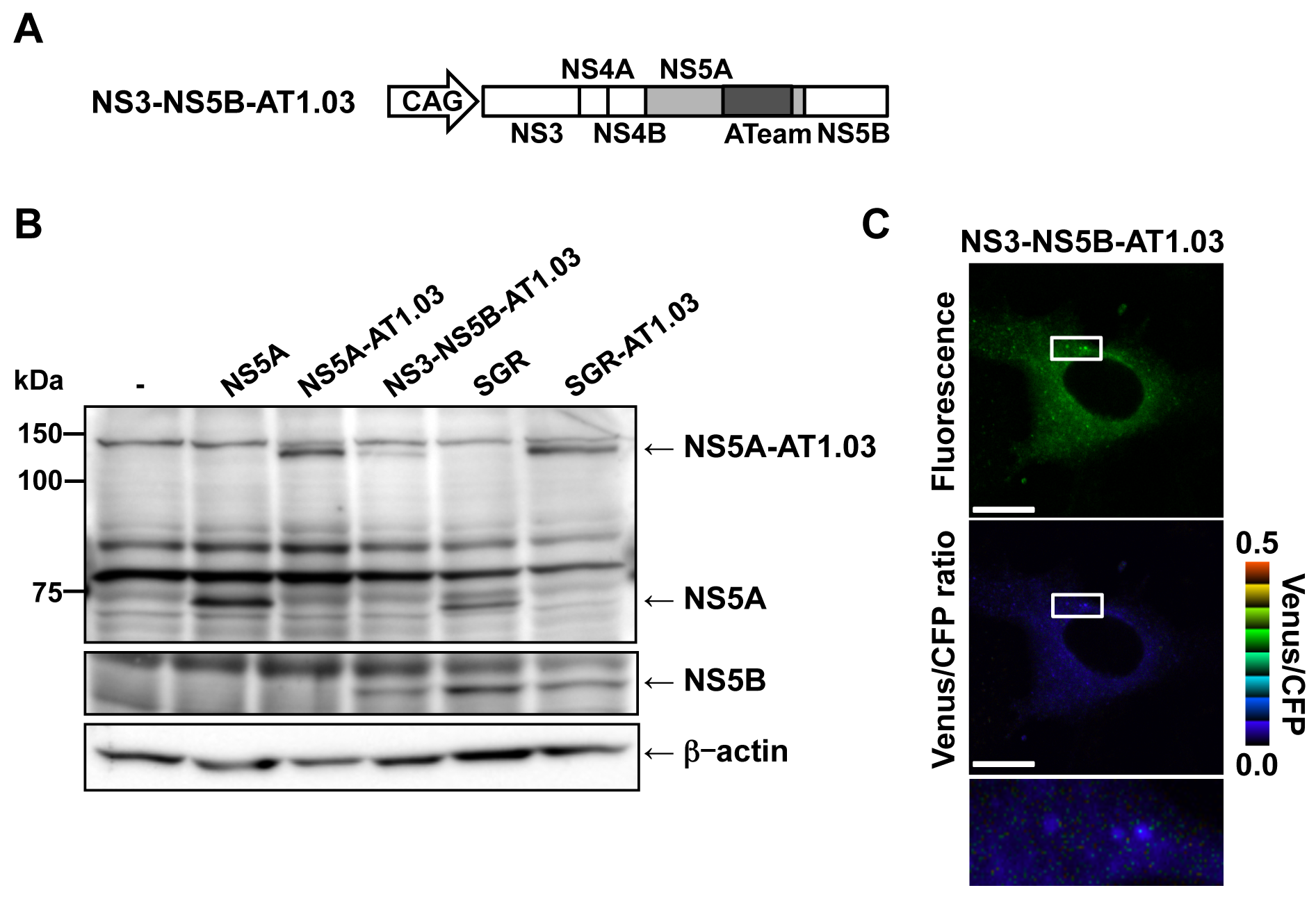

Supplement: Figure S4 — Visualization of the ATP level in cells expressing replication-defective HCV polyprotein. (A) A schematic representation of the NS3-NS5B-AT1.03 plasmid is shown. The HCV polyprotein is indicated by the open boxes. The ATeam gene was inserted into the same site as that for NS5A-ATeam and SGR-ATeam insertion as indicated in the legend for Figure 4A. CAG, CAG promoter. (B) Cells transfected with constructs encoding NS5A, NS5A-AT1.03, NS3-NS5B-AT1.03, SGR or SGR-AT1.03 were analyzed by immunoblotting with anti-NS5A, anti-NS5B or anti-beta-actin antibodies. (C) Huh-7 cells were transfected with NS3-NS5B-AT1.03, and analyzed in the same way as described in the legends for Figures 5A and 5B. The upper panel (Fluorescence) demonstrates signal intensity from a spectral channel with maximum intensity and represents the expression pattern of NS5A-ATeam processed from NS3-NS5B-AT1.03. The lower panels (Venus/CFP ratio) indicate the FRET ratio and a five-fold magnification of the boxed area. Scale bar, 20 µm. (TIF) [file ppat.1002561.s004.tif]

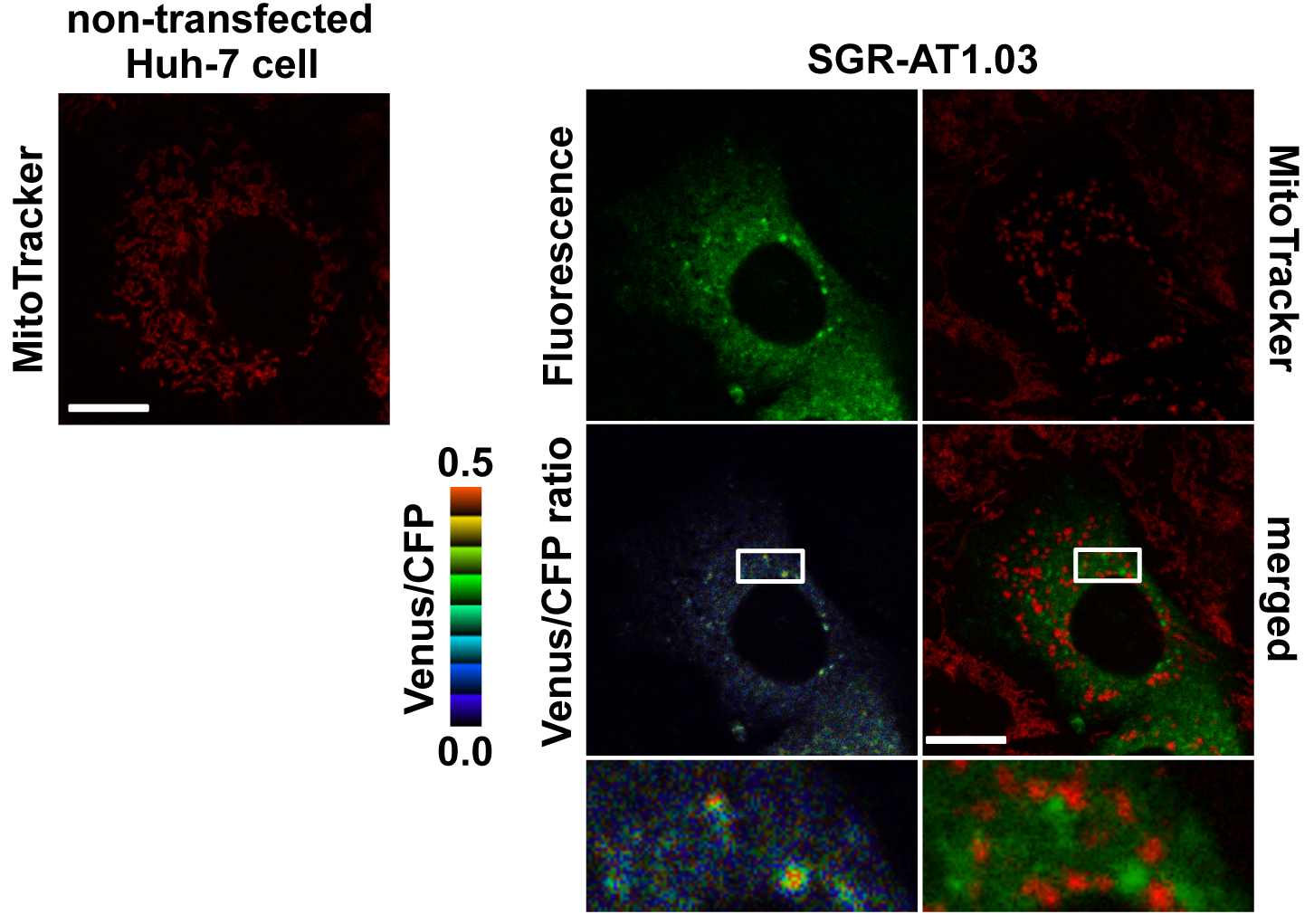

Supplement: Figure S5 — Relationship between ATP-enriched dot-like structures and mitochondria. Huh-7 cells replicating SGR-AT1.03 (right panels) and parental cells (left panel) were analyzed. Active mitochondria were labeled with MitoTracker Red CMXRos in living cells, and were analyzed in the same way as described in the legends for Figures 5A and 5B, using a reference for the MitoTracker spectrum. The lowest panels of SGR-ATeam cells indicate five-fold magnifications of the boxed areas. Scale bars, 20 µm. (TIF) [file ppat.1002561.s005.tif]

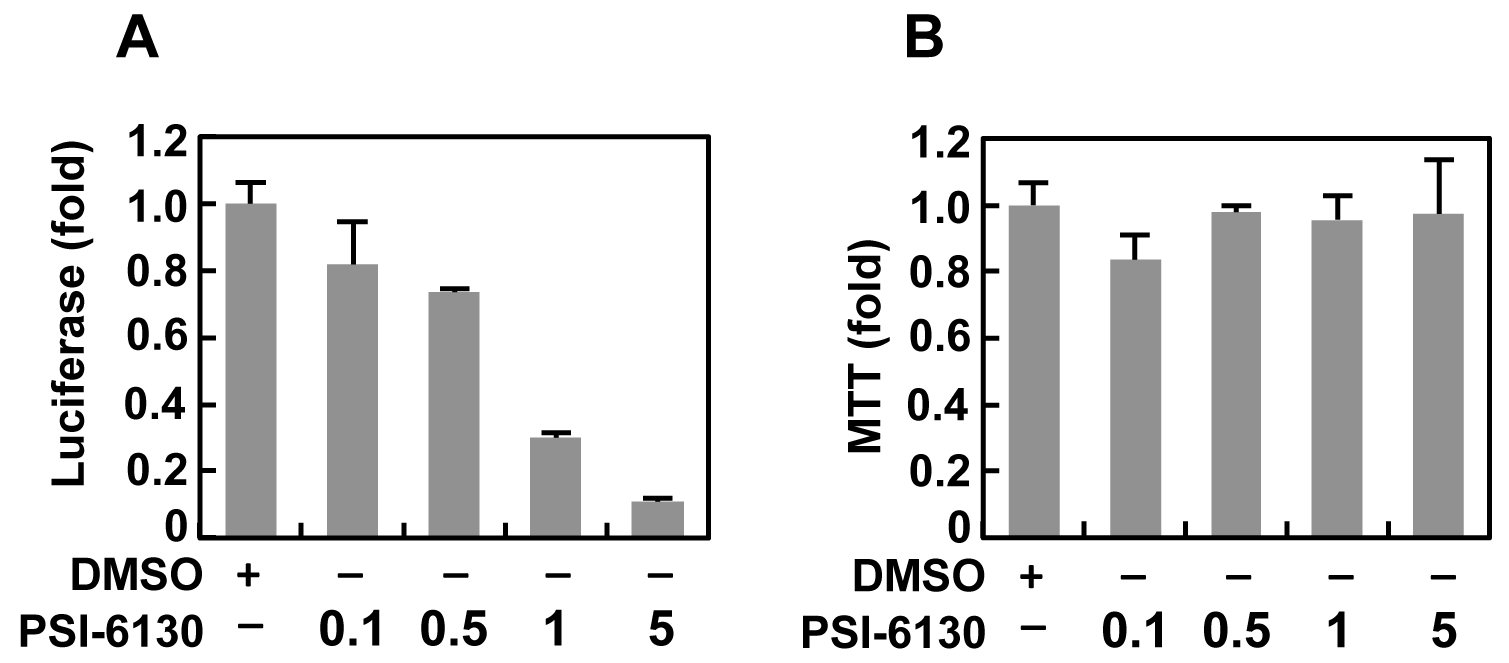

Supplement: Figure S6 — Inhibitory effect of PSI-6130 on HCV RNA replication. (A) Replication levels of SGR/luc-AT1.03 RNA in transfected cells were determined by luciferase assay 3 days after treatment with PSI-6130 at the indicated concentrations (µM). The values shown were normalized for transfection efficiency with luciferase activity determined 24 h post-transfection. All data are presented as means and SD for three independent samples. (B) Cell viability was assessed using the MTT assay. (TIF) [file ppat.1002561.s006.tif]
